# Infant feeding and health-related quality of life in healthy Chinese infants: results from a prospective, observational cohort study

**DOI:** 10.1186/s12955-016-0518-3

**Published:** 2016-08-08

**Authors:** Nicholas P. Hays, Meng Mao, Lan Zhang, John Ge, Robert Northington, Manjiang Yao, Sheri Volger

**Affiliations:** 1 Nestlé Nutrition R&D, King of Prussia, PA USA; 2 Chengdu Women’s and Children’s Central Hospital, Chengdu, China; 3 Wyeth Nutritional Company Ltd, Shanghai, China

**Keywords:** Breastfeeding, Infant formula, Infant and Toddler Quality of Life Questionnaire (ITQOL), Short Form 12 Health Survey, version 2 (SF-12v2), Patient outcome assessment

## Abstract

**Background:**

Infant feeding regimens, including breastfeeding, formula-feeding, or a combination of the two, may influence infant health-related quality of life (HRQOL). However, few studies have examined this association.

**Methods:**

This prospective cohort study assessed HRQOL in relation to three parent-selected feeding regimens: exclusively breastfed (*n* = 136), exclusively study formula-fed (*n* = 140), and mixed-fed with study formula and breast milk (*n* = 151). Healthy Chinese infants were enrolled at their first normally scheduled well infant clinic visit at age 42 days (study day 1). Parents independently chose their infants’ feeding regimens prior to recruitment into the study, with infants in the formula and mixed-fed groups already consuming an infant formula enriched with α-lactalbumin and increased *sn*-2 palmitate and oligofructose. The Infant and Toddler Quality of Life Questionnaire, which includes six infant-focused and three parent-focused concepts, was used to assess HRQOL at day 1 and at a follow-up visit 48 days later. Scores for each concept ranged from 0 to 100. Parent quality of life (assessed using the Mental Component Summary score of the SF-12v2 Health Survey) was included in the ANCOVA model to adjust for its potential effect on parent’s perception of infant HRQOL.

**Results:**

HRQOL concept scores were high in all three study groups at both visits (mean scores 71–95). Day 1 HRQOL scores were not significantly different between groups. At day 48, 5 of 9 HRQOL scores were not significantly different between groups. However, scores for Temperament and Moods, General Health Perceptions and Parent Impact–Time were slightly but statistically significantly lower in the formula-fed group (mean scores 75–86; all *p* ≤ 0.01) compared to the breastfed (78–90) and mixed-fed (77–91) groups. Day 48 Parent Impact–Emotional scores were also significantly lower by a small margin (4 points; *p* = 0.003) in the formula-fed group compared with the breastfed group.

**Conclusions:**

HRQOL was high in this population of healthy infants, with only a few small differences in HRQOL concept scores observed between breastfed, formula-fed and mixed-fed infants. These results indicate favorable physical, mental, and social well-being in these infants and parents. Assessment of infant HRQOL is therefore feasible and provides valuable insight into parental perceptions of their child’s health and well-being.

**Trial registration:**

ClinicalTrials.gov, NCT01370967.

## Background

Breast milk is considered the best source of nutrition for infants [1], but for a variety of reasons many parents choose to supplement breastfeeding with some formula-feeding or to provide feedings exclusively with infant formula. Optimizing health outcomes of formula-fed infants is therefore a public health priority [2]. Infant health-related quality of life (HRQOL), a broad concept that encompasses aspects of physical, psychological, and social function [3], is an important outcome that may be directly affected by an infant’s feeding regimen in at least two ways. First, the infant’s feeding regimen may influence feeding tolerance and gastrointestinal (GI) symptoms. For example, compared with breastfed infants, infants who are fed standard formula have harder stools [4, 5], which can lead to constipation and discomfort. Second, feeding regimen may influence immune function and susceptibility to illnesses or infections [6]. For example, breast milk has been shown to support immunity through transmission of maternal immune agents and by promoting a unique balance of microflora in the gut [7] that may offer immune benefits [8]. However, despite a need to better understand infant HRQOL in relation to feeding regimen, there is little research on this association to date [9].

Infant formula containing structured lipid (i.e., fat with an increased proportion of palmitic acid in the *sn*-2 position) and oligofructose (a non-digestible soluble dietary fiber) has been developed to promote the formation of softer stools that are more like those of breastfed infants [10, 11]. In addition, oligofructose is a prebiotic [12] that has been shown to influence the growth of bifidobacteria to levels that are within the range of levels found in breastfed infants [13]. The aim of this study was to assess HRQOL in infants consuming an infant formula with increased *sn*-2 palmitate and oligofructose (study formula) either alone or as a supplement to breastfeeding, and to compare this to HRQOL in infants consuming breast milk exclusively. Although there are methodologic difficulties of measuring HRQOL in infants [14], several valid and reliable instruments exist to examine infant HRQOL through proxy report by the parent or caregiver [9, 15–17]. We used the Infant and Toddler Quality of Life Questionnaire (ITQOL) [17] to assess HRQOL as a function of feeding regimen in a large prospective study of Chinese infants. We hypothesized that infants fed the study formula either alone or as a supplement to breast milk would have the same HRQOL as infants fed breast milk alone.

## Methods

### Participants

This was a 48-day prospective, observational cohort study involving a representative sample of healthy term infants enrolled from 24 hospitals across 14 major cities in Eastern, Central and Western China between September 2011 and June 2013. The study was approved by the Institutional Ethics Committee of each hospital. Infants 35–49 days old (mean 42 days) were enrolled at the time of their normally scheduled, 6-week, well infant clinic visit and grouped based on their parent-selected, pre-study feeding regimen. Infants were included if they were healthy, singleton, born at 37–42 weeks of gestation, and measured between the 5th and 95th percentiles (inclusive) in weight-for-age according to the World Health Organization (WHO) growth standards [18]. Infants with major congenital anomalies, suspected or documented systemic or congenital infections, or other severe acute or chronic medical conditions or laboratory abnormalities that would have increased the risk associated with study participation or interfered with interpretation of results were excluded. Likewise, infants with conditions requiring infant feedings other than those specified in the protocol, those receiving complementary foods or liquids (e.g., more than 5 mL of fruit/vegetable juice per day), or those receiving any medication(s) or vitamin/mineral/herbal supplement(s) known or suspected to affect study outcomes (e.g., fat digestion, absorption and/or metabolism; stool characteristics) were excluded. Finally, infants were included in the study only if parent(s) or legally acceptable representative(s) (henceforth “parents”) provided informed consent indicating that they were willing and able to comply with scheduled visits and other study procedures.

### Measures

The study included data collection at three clinic visits: clinic visit 1 (study day 1; infant age ~42 days), clinic visit 2 (study day 18 ± 3; infant age ~60 days), and clinic visit 3 (study day 48 ± 3; infant age ~90 days). The timing of data collection was chosen to coincide with the typical schedule of well infant clinic visits in China. Feeding regimen was assessed at clinic visit 1, and HRQOL data were collected at clinic visits 1 and 3.

#### Exposure: feeding regimen

Infants were grouped into three different categories based on the infants’ feeding regimens, previously chosen by parents, and fed for a period of at least 3 days prior to enrollment: (1) exclusively study formula-fed, (2) exclusively breastfed, or (3) mixed-fed with study formula and breast milk. To be included in a particular feeding group, parent(s) must have previously made the decision to voluntarily continue with their infant’s current feeding regimen. Infants fed any types or brands of formula other than the study formula (either exclusively or in a mixed feeding regimen) at the time of study enrollment were excluded. Study formula was commercially available and thus fully compliant with Chinese infant formula standards, and was not provided to any of the study participants; rather, parents continued to purchase the formula as they had prior to study enrollment. Investigators were not involved in any feeding decisions.

Parents were free to switch their infant to other feeding regimens at any time during the study. Continued follow-up of these infants depended on the new feeding regimen and the timing of the switch relative to study days 15–17 (when parents completed the second of several three-day diaries on infant stool characteristics, another study outcome reported separately [19]). If the infant was switched to another of the three study feeding regimens (e.g., from exclusively breastfed to mixed-fed with study formula and breast milk) *before* the completion of the second three-day infant stool diary, the infant continued to participate in the study. If the infant was switched from their current feeding regimen to a feeding regimen with a non-study formula *before* the completion of the second three-day infant stool diary, the infant was withdrawn from the study. If the infant was switched to any feeding regimen after the second three-day infant stool diary, the infant remained in the study. Infants remained categorized according to their initial feeding regimen, even if switching occurred later. Only 38 infants switched to a different feeding regimen during the study.

#### Outcome: health-related quality of life

Infant HRQOL was assessed using a standardized validated questionnaire, the Infant and Toddler Quality of Life Questionnaire (ITQOL), at clinic visit 1 and clinic visit 3. The ITQOL was translated for this study into Simplified Chinese using a standardized linguistic validation process, and the translated tool has been shown to perform well, have good reliability, and discriminate across illness-related categories in this population of very young infants [20].

The ITQOL questionnaire consists of 97 questions. Of these, 29 are not relevant for infants who are less than 1 year old; thus this study used the remaining 68 questions. The ITQOL asks parents about six infant-focused concepts including overall health, physical abilities, growth and development, bodily pain/discomfort, temperament and moods, and general health perceptions. In addition, the ITQOL measures three parent-focused concepts including the impact of infant health and well-being on parents’ emotions, such as worry (“Parent Impact—Emotional”), on parents’ time for personal needs (“Parent Impact—Time”), and on parents’ perceptions of how the family is getting along with one another (“Family Cohesion”). The recall period for the ITQOL is 4 weeks. For each of the six infant-focused and three parent-focused concepts, the mean response values for items used to assess the concept constituted the raw score. In cases of missing values, the mean was calculated on non-missing data if fewer than half of the concept items were missing; if half or more were missing then the concept value was set to missing. Raw scores were standardized to the range of possible concept scores using standardized scoring instructions [21]. The standardized values ranged from 0 to 100, with higher scores indicating better quality of life for the particular concept being measured.

#### Covariate: parent health-related quality of life

Parent HRQOL was assessed using the Short Form 12 Health Survey, version 2 (SF-12v2) [22]. The Chinese version of this questionnaire has been shown to be valid, reliable and sensitive for this population [23]. The same caregiver / parent completed both the SF-12v2 and the ITQOL. The SF-12v2 assesses 8 health domains from which two summary measures are calculated: the Physical Component Summary (PCS) and the Mental Component Summary (MCS). If more than half of the questions within a health domain was missing, then that health domain was considered missing and the PCS and MCS were not calculated. Health domain scores were standardized to a mean of 50 and a standard deviation of ten with higher scores indicating better health [24]. The MCS score of the SF-12v2 was used to adjust for potential effects of the parent’s own mental functioning on their perception of their infant’s quality of life.

### Statistical analyses

Analysis of covariance (ANCOVA) was used to compare the scores for the six infant-focused concepts and three parent-focused concepts of HRQOL across the three feeding regimen groups at day 1 and day 48. Since infants were enrolled based on their pre-existing feeding regimens, scores at day 1 cannot be considered baseline values and thus were not included in the day 48 ANCOVA model as a covariate. ITQOL concept scores were found to be non-normally distributed, therefore a non-parametric alternative to ANCOVA based on ranks [25] was performed for the main analysis; parametric tests were performed for comparison and found to produce similar results (data not shown). The MCS score from the SF-12v2 was included in the model as a covariate. An overall comparison of the three feeding groups was done as well as the three possible pairwise comparisons. Analyses were conducted using SAS (version 9.1.3, SAS Institute Inc., Cary, NC, USA).

## Results

**Fig. 1:**
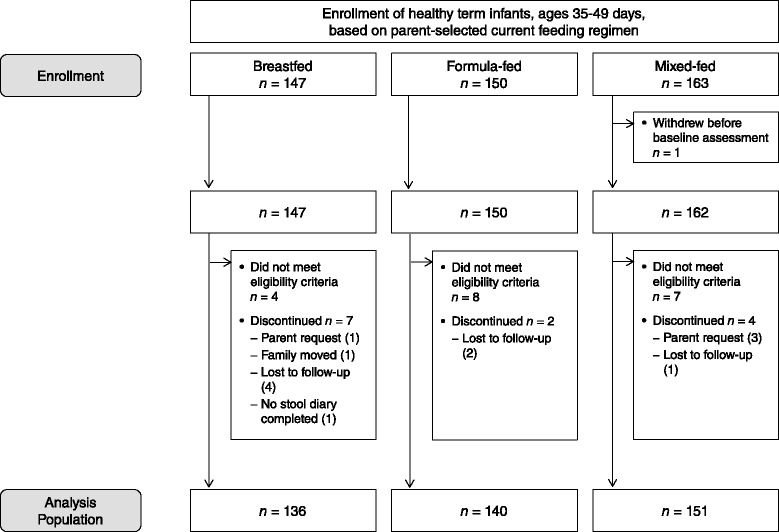
Enrollment and discontinuation of study participants (Breastfed, group of infants who were exclusively fed breast milk; Formula-fed, group of infants who were exclusively fed with formula containing high *sn*-2 palmitate and oligofructose; Mixed-fed, group of infants who were breastfed and fed with formula containing high *sn*-2 palmitate and oligofructose)

The study included 136 exclusively breastfed, 140 exclusively study formula-fed, and 151 mixed-fed infants (Fig. 1). Feeding regimen groups were similar with regard to age at study enrollment, sex, and ethnicity (Table 1). Exclusively study formula-fed infants were born at a slightly earlier gestational age, on average, than exclusively breastfed or mixed-fed infants. All study infants were Asian and none attended daycare during the study. Infants in the mixed-fed group had a generally stable feeding profile over the study interval (percentage of daily feedings as breastfeeds = 58.3 % ± 20.1 % at study day 1 and 58.1 % ± 19.8 % at day 48). In regard to parental characteristics, mothers and fathers of study formula-fed infants had slightly but significantly fewer years of education compared to the other groups, with no differences in occupational status (Table 1). There was no difference (*p* = 0.48) in monthly household income among the three groups. The average number of adults living in the household was high and did not differ among groups (3.1 ± 1, *p* = 0.96). Similarly, the proportion of infants delivered by Cesarean section was high (~55 %) and not different across groups (*p* = 0.21).

**Table 1:** Infant, parent, and household characteristics at time of study enrollment ^a^

Characteristics	Study formula-fed only (*n* = 140)	Breastfed only (*n* = 136)	Mixed-fed (breastfed and study formula-fed) (*n* = 151)	*P*-value^b^
Infants				
Age, days	42.2 ± 3.6	42.3 ± 3.7	42.5 ± 3.4	0.75
Gestational age, weeks	38.6 ± 1.0	39.0 ± 1.0	38.9 ± 1.0	0.004
Weight, kg^c^	4.9 ± 0.5	5.0 ± 0.5	5.0 ± 0.5	ND
Sex, % male	70 (50.0)	78 (57.4)	93 (61.6)	0.13
Birth order, % first birth	116 (82.9)	122 (89.7)	129 (85.4)	0.34
Type of delivery, % Cesarean	82 (58.6)	66 (48.5)	85 (56.3)	0.21
Attend day care	0	0	0	1.00
Mothers				
Education, years	14.6 ± 2.8	15.8 ± 3.0	15.3 ± 2.8	0.002
Occupation				0.53
Professional	42 (30.0)	51 (37.5)	55 (36.4)	
Service or retail	23 (16.4)	21 (15.4)	22 (14.6)	
Homemaker	32 (22.9)	17 (12.5)	27 (17.9)	
Fathers				
Education, years	14.7 ± 3.0	15.6 ± 2.8	15.4 ± 3.0	0.031
Occupation				0.28
Professional	25 (17.9)	28 (20.6)	30 (19.9)	
Service or retail	31 (22.1)	34 (25.0)	34 (22.5)	
Technician/associate professional	22 (15.7)	25 (18.4)	21 (13.9)	

^a^Data presented as mean ± standard deviation or number (%); *ND* not determined

^b^ *p*-values from ANOVA (continuous variables) or Fisher’s Exact or Chi-Square tests (categorical variables)

^c^Weight presented for safety population (*n* = 148 for study formula-fed only, *n* = 137 for breastfed only, and *n* = 155 for mixed-fed group)

Parent SF-12v2 summary scores, which were assessed as a covariate in this study, increased from day 1 to day 48 and differed only slightly across feeding regimen groups (Table 2). The MCS score was shown to be moderately positively correlated with all ITQOL parent-focused concepts at day 1 (all r ≥ 0.35, *p* < 0.05, Appendix) and at day 48 (all r ≥ 0.37, *p* < 0.05, Appendix).

**Table 2:** Parent SF-12v2 Physical Component Summary (PCS) and Mental Component Summary (MCS) scores across infant feeding regimen groups at enrollment (study day 1) and study day 48

Subscale	Study formula-fed only	Breastfed only	Mixed-fed (breastfed and study formula-fed)
PCS, mean ± SD			
Day 1	49.2 ± 8.1	50.6 ± 6.6	49.9 ± 7.7
Day 48	51.6 ± 6.6	52.6 ± 5.4	51.7 ± 7.2
MCS, mean ± SD			
Day 1	50.3 ± 8.9	51.8 ± 8.0	50.7 ± 8.2
Day 48	53.1 ± 7.1	52.1 ± 8.4	52.2 ± 7.9

Comparisons of ITQOL concept scores across feeding regimen groups revealed no significant differences at day 1 (Table 3). On day 48, the study formula-fed group was found to be significantly lower than both the breastfed group and the mixed-fed groups on the Temperament and Moods score, the General Health Perceptions score, and the Parent Impact—Time score (Table 3). Study formula-fed infants were also found to be significantly lower than breastfed infants (but not mixed-fed infants) on the Parent Impact—Emotional score at day 48 (Table 3). These differences ranged from 3 to 5 points on scores standardized to range from 0 to 100.

**Table 3:** ITQOL scores across infant feeding regimen groups at enrollment (study day 1) and study day 48^a^

	Study formula-fed only (*n* = 140)	Breastfed only (*n* = 136)	Mixed-fed (breastfed and study formula-fed) (*n* = 151)	*P*-value^b^
Infant-focused concepts				
Overall Health				
Day 1	77.9 ± 19.2	80.0 ± 15.9	78.4 ± 17.4	0.932
Day 48	79.4 ± 16.4	78.6 ± 14.5	79.3 ± 17.6	0.623
Physical Abilities				
Day 1	94.3 ± 6.3	86.4 ± 15.6	80.6 ± 28.3	0.878
Day 48	94.1 ± 6.3	84.7 ± 18.6	75.9 ± 29.8	0.410
Growth and Development				
Day 1	81.4 ± 15.2	84.3 ± 14.1	82.3 ± 14.6	0.405
Day 48	84.9 ± 13.9	84.5 ± 14.6	83.6 ± 15.3	0.845
Bodily Pain/Discomfort				
Day 1	92.9 ± 12.6	93.6 ± 11.7	92.9 ± 12.4	0.877
Day 48	94.1 ± 11.4	94.9 ± 8.8	94.3 ± 9.8	0.920
Temperament and Moods				
Day 1	71.2 ± 8.0	73.8 ± 9.5	72.2 ± 9.1	0.127
Day 48	74.5 ± 9.2 ^1^	77.7 ± 8.4 ^2^	77.0 ± 8.3 ^2^	0.0057
General Health Perceptions				
Day 1	81.3 ± 11.4	84.2 ± 10.2	83.3 ± 10.5	0.172
Day 48	81.1 ± 11.7 ^1^	84.7 ± 11.8 ^2^	85.5 ± 9.3 ^2^	0.0009
Parent-focused concepts				
Parent Impact – Emotional				
Day 1	87.6 ± 17.1	89.4 ± 17.0	87.0 ± 15.4	0.324
Day 48	88.0 ± 15.7 ^1^	91.9 ± 12.8 ^2^	90.0 ± 10.8 ^1,2^	0.009
Parent Impact – Time				
Day 1	83.3 ± 21.4	88.2 ± 15.4	88.1 ± 13.3	0.456
Day 48	85.9 ± 18.7 ^1^	90.0 ± 14.2 ^2^	90.9 ± 10.8 ^2^	0.0053
Family Cohesion				
Day 1	79.9 ± 19.5	79.5 ± 18.6	79.3 ± 18.0	0.855
Day 48	81.0 ± 16.2	79.0 ± 18.4	80.6 ± 19.1	0.649

^a^Data presented as unadjusted mean ± standard deviation

^b^Main effects *p*-value from non-parametric ANCOVA (parent Mental Component Summary [MCS] score from the SF-12v2 included as a covariate)

^1,2^Values that do not share the same superscript are significantly different (all pairwise *p* < 0.01)

## Discussion

In this study, we found high HRQOL scores at both study visits, regardless of feeding regimen. A few small differences were observed at day 48 between study formula-fed and exclusively breastfed infants on two infant-focused HRQOL concepts (Temperament and Moods, General Health Perceptions) and two parent-focused HRQOL concepts (Parent Impact—Time and Parent Impact—Emotional). Similar small differences were observed at day 48 between the study formula-fed and mixed-fed groups for Temperament and Moods, General Health Perceptions, and Parent Impact—Time. All of these differences, while statistically significant, were quite modest – ranging from 3 to 5 points on a 100-point scale. Prior studies consistently suggest that changes in HRQOL of a half a standard deviation or greater represent meaningful changes in people’s experiences; this appears to hold across a variety of measures [26]. We observed ITQOL concept score differences of between a third and a half a standard deviation between feeding regimen groups, differences that are below this clinically meaningful threshold. Although these differences may not be clinically relevant, it is possible that other unmeasured factors such as maternal stress, postpartum depression, sleep quality, or other social determinants of health in the study formula-fed group may have contributed to both the parents’ initial choice of feeding regimens and the slightly lower Parent Impact—Emotional and Parent Impact—Time scores in the study formula-fed group. Likewise, Temperament and Moods may have been lower in the formula-fed group due to the parent’s rationale for initiating formula-feedings or other infant health measures, which this study was not designed to assess. The observed differences in HRQOL may also be due to differences in infant feeding mode (bottle vs. breastfeeding) rather than nutrition (formula or breast milk) or other unmeasured factors.

In general, HRQOL scores for our study population were high, indicating good overall health and well-being in this sample. For comparison, a study in infants 13 months of age with severe asthma-like symptoms reported mean ITQOL concept scores of 61 for Overall Health, 70 for Temperament and Moods, and 66 for Bodily Pain [27]. In addition, the mean SF-12v2 scores of the parents of infants in the present study were close to the expected values for a general adult population (scores are norm-based on the US general population with a mean score of 50 and a standard deviation of ten; scores in Chinese adults have been shown to be very similar [23, 28]). A variety of health-promoting factors contribute to infant well-being, and although decisions about infant feeding are important, their impact may be attenuated in the presence of these other factors. For example, social factors may influence infant well-being (real and/or perceived), and the mean number of adults in the household of infants in this study (~3) suggests a high caregiver-to-infant ratio which may have contributed to high HRQOL scores. Less healthy infants or those in less favorable social conditions may be more sensitive than our study population to the potential effects of feeding regimens on HRQOL. Hence, future research should evaluate the relationship between feeding regimens and HRQOL in other infant populations, especially those with lower HRQOL.

Self-assessed HRQOL is impossible for infants due to their young age. Therefore infant HRQOL can be assessed only by proxy, typically by parental or caregiver report. However the quality of life of the proxy may influence their perception of the infant’s quality of life. In order to address this potential bias, we included maternal quality of life (assessed using SF-12v2 MCS score) as a covariate in the analysis of ITQOL scores across feeding groups. This approach may be broadly applicable to other studies of infant HRQOL, and the use of a single global summary score such as the MCS may be preferred over assessing multiple individual factors (e.g. family income, occupation, and educational level) that may influence parental rating of infant HRQOL.

Our study adds to a limited body of literature assessing the impact of feeding regimen on quality of life in infants. Most published studies have assessed the impact of breastfeeding on parental quality of life and have not evaluated effects on infant quality of life [29–31]. Manificat and Dazord [32] examined quality of life among 105 infants (mean age 5.7 months) as a function of feeding, using the Quality of Life of the Infant Scale [9] and reported that maternal overall perception of infant quality of life was independent of the mode of feeding. However, duration of breastfeeding greater than 3 months was associated with higher maternal-reported scores on four items, including infant nervousness, infant anxiousness, family cohesiveness, and infant total quality of life (visual analogue scale) [32]. This finding of differences on some, but not all, items is similar to our results.

Strengths of our study include use of the ITQOL, which was chosen based on extensive review of alternative measures. We chose the ITQOL over other measures [9, 15] because it has been most extensively used, is appropriate for measurement of HRQOL in healthy infants [20], and provides clearly defined concept scores. The inclusion of a mixed-fed group is an additional strength; most studies exclude this group despite widespread use of mixed feeding regimens and practitioner encouragement of partial breastfeeding when exclusive breastfeeding is not possible [33]. Finally, assessment of HRQOL in both parents and infants is important for appropriate adjustment for the potential confounding influence of parental HRQOL; the correlations between the SF-12v2 and the ITQOL parent-focused concepts highlight the importance of assessing both of these factors.

Limitations of our study include potential lack of generalizability to the overall population of Chinese infants given the high educational level of study parents, inclusion of infants consuming only one type of infant formula, and high HRQOL of study infants. There is also potential for residual confounding in our observational study due to unmeasured factors that influence both choice of feeding regimen and HRQOL. In addition, HRQOL was reported by parents not blinded to feeding regimen; it is possible that reporting of HRQOL was influenced by parents’ beliefs about the effects of feeding regimens on infant HRQOL, rather than based purely on observations of the infant’s well-being. Despite these limitations, our observational approach had the advantage of assessing feeding regimens and their outcomes in real-world settings.

## Conclusion

Our study represents an important contribution to the limited literature on infant HRQOL, and highlights the feasibility and utility of measuring HRQOL in this population. We found high HRQOL concept scores in this population of healthy infants, regardless of which feeding regimen parents had chosen. Only a few small differences in HRQOL concept scores were observed between the breastfed, study formula-fed and mixed-fed infants and the effect size of these differences suggests they may be of limited clinical relevance. Furthermore, no significant differences were observed between groups in Overall Health, Physical Abilities, Growth and Development, or Bodily Pain/Discomfort scores, areas where nutrition might be expected to play a role. Based on these results, we conclude that, in this sample of healthy Chinese infants, there was little to no effect of the specific feeding regimens assessed in this study on infant HRQOL. Future research should examine relationships between feeding regimens and HRQOL in other infant populations including those with lower overall health and well-being.

## Abbreviations

ANCOVA, analysis of covariance; GI, gastrointestinal; HRQOL, health-related quality of life; ITQOL, infant and toddler quality of life questionnaire; MCS, mental component summary; PCS, physical component summary; SF-12v2, short form 12 health survey, version 2; WHO, World Health Organization

## Appendix

**Table 4:** Pearson correlations between Mental Component Summary (MCS) and Physical Component Summary (PCS) scores from SF-12v2 and Parent Impact – Emotional (PE), Parent Impact – Time (PT) and Family Cohesion (FC) scores from the ITQOL, study day 1

	MCS	PCS	PE	PT	FC
Mental Component Summary (MCS)					
Correlation Coefficient	1.00	**0.12**	**0.38**	**0.41**	**0.35**
Physical Component Summary (PCS)					
Correlation Coefficient	**0.12**	1.00	0.05	**0.20**	**0.19**
Parent Impact – Emotional (PE)					
Correlation Coefficient	**0.38**	0.05	1.00	**0.39**	**0.12**
Parent Impact – Time (PT)					
Correlation Coefficient	**0.41**	**0.20**	**0.39**	1.00	**0.24**
Family Cohesion (FC)					
Correlation Coefficient	**0.35**	**0.19**	**0.12**	**0.24**	1.00

Correlation coefficients in bold are statistically significant at *p* < 0.05

**Table 5:** Pearson correlations between Mental Component Summary (MCS) and Physical Component Summary (PCS) scores from SF-12v2 and Parent Impact – Emotional (PE), Parent Impact – Time (PT) and Family Cohesion (FC) scores from the ITQOL, study day 48

	MCS	PCS	PE	PT	FC
Mental Component Summary (MCS)					
Correlation Coefficient	1.00	**0.17**	**0.44**	**0.37**	**0.40**
Physical Component Summary (PCS)					
Correlation Coefficient	**0.17**	1.00	**0.30**	**0.31**	**0.26**
Parent Impact – Emotional (PE)					
Correlation Coefficient	**0.44**	**0.30**	1.00	**0.42**	**0.27**
Parent Impact – Time (PT)					
Correlation Coefficient	**0.37**	**0.31**	**0.42**	1.00	**0.16**
Family Cohesion (FC)					
Correlation Coefficient	**0.40**	**0.26**	**0.27**	**0.16**	1.00

Correlation coefficients in bold are statistically significant at *p* < 0.05
